# Racial and Ethnic Differences in Emergency Department Diagnostic Imaging at US Children’s Hospitals, 2016-2019

**DOI:** 10.1001/jamanetworkopen.2020.33710

**Published:** 2021-01-29

**Authors:** Jennifer R. Marin, Jonathan Rodean, Matt Hall, Elizabeth R. Alpern, Paul L. Aronson, Pradip P. Chaudhari, Eyal Cohen, Stephen B. Freedman, Rustin B. Morse, Alon Peltz, Margaret Samuels-Kalow, Samir S. Shah, Harold K. Simon, Mark I. Neuman

**Affiliations:** 1 Department of Pediatrics, UPMC Children’s Hospital of Pittsburgh, Pittsburgh, Pennsylvania; 2 Department of Radiology, UPMC Children’s Hospital of Pittsburgh, Pittsburgh, Pennsylvania; 3 Children’s Hospital Association, Lenexa, Kansas; 4 Division of Emergency Medicine, Department of Pediatrics, Ann & Robert H. Lurie Children’s Hospital of Chicago, Northwestern University Feinberg School of Medicine, Chicago, Illinois; 5 Section of Pediatric Emergency Medicine, Departments of Pediatrics and Emergency Medicine, Yale School of Medicine, New Haven, Connecticut; 6 Division of Emergency and Transport Medicine, Children’s Hospital Los Angeles, Keck School of Medicine of the University of Southern California, Los Angeles; 7 Department of Pediatrics, The Hospital for Sick Children, Toronto, Ontario, Canada; 8 Sections of Pediatric Emergency Medicine and Gastroenterology, Department of Pediatrics, Alberta Children’s Hospital, Alberta Children’s Hospital Research Institute, Cumming School of Medicine, University of Calgary, Calgary, Alberta, Canada; 9 Department of Emergency Medicine, Alberta Children’s Hospital, Alberta Children’s Hospital Research Institute, Cumming School of Medicine, University of Calgary, Calgary, Alberta, Canada; 10 Department of Pediatrics, Nationwide Children’s Hospital, Columbus, Ohio; 11 Department of Population Medicine, Harvard Medical School, Harvard Pilgrim Health Care Institute, Boston, Massachusetts; 12 Department of Emergency Medicine, Massachusetts General Hospital, Boston; 13 Divisions of Hospital Medicine and Infectious Diseases, Department of Pediatrics, Cincinnati Children’s Hospital Medical Center, Cincinnati, Ohio; 14 Division of Emergency Medicine, Departments of Pediatrics and Emergency Medicine, Emory University School of Medicine, Children’s Healthcare of Atlanta, Atlanta, Georgia; 15 Division of Emergency Medicine, Boston Children’s Hospital, Harvard Medical School, Boston, Massachusetts

## Abstract

**Importance:**

Diagnostic imaging is frequently performed as part of the emergency department (ED) evaluation of children. Whether imaging patterns differ by race and ethnicity is unknown.

**Objective:**

To evaluate racial and ethnic differences in the performance of common ED imaging studies and to examine patterns across diagnoses.

**Design, Setting, and Participants:**

This cross-sectional study evaluated visits by patients younger than 18 years to 44 US children’s hospital EDs from January 1, 2016, through December 31, 2019.

**Exposures:**

Non-Hispanic Black and Hispanic compared with non-Hispanic White race/ethnicity.

**Main Outcomes and Measures:**

The primary outcome was the proportion of visits for each race/ethnicity group with at least 1 diagnostic imaging study, defined as plain radiography, computed tomography, ultrasonography, and magnetic resonance imaging. The major diagnostic categories classification system was used to examine race/ethnicity differences in imaging rates by diagnoses.

**Results:**

A total of 13 087 522 visits by 6 230 911 children and adolescents (mean [SD] age, 5.8 [5.2] years; 52.7% male) occurred during the study period. Diagnostic imaging was performed during 3 689 163 visits (28.2%). Imaging was performed in 33.5% of visits by non-Hispanic White patients compared with 24.1% of visits by non-Hispanic Black patients (odds ratio [OR], 0.60; 95% CI, 0.60-0.60) and 26.1% of visits by Hispanic patients (OR, 0.66; 95% CI, 0.66-0.67). Adjusting for confounders, visits by non-Hispanic Black (adjusted OR, 0.82; 95% CI, 0.82-0.83) and Hispanic (adjusted OR, 0.87; 95% CI, 0.87-0.87) patients were less likely to include any imaging study compared with visits by non-Hispanic White patients. Limiting the analysis to only visits by nonhospitalized patients, the adjusted OR for imaging was 0.79 (95% CI, 0.79-0.80) for visits by non-Hispanic Black patients and 0.84 (95% CI, 0.84-0.85) for visits by Hispanic patients. Results were consistent in analyses stratified by public and private insurance groups and did not materially differ by diagnostic category.

**Conclusions and Relevance:**

In this study, non-Hispanic Black and Hispanic children were less likely to receive diagnostic imaging during ED visits compared with non-Hispanic White children. Further investigation is needed to understand and mitigate these potential disparities in health care delivery and to evaluate the effect of these differential imaging patterns on patient outcomes.

## Key Points

**Question:**

Does the use of diagnostic imaging for children receiving care in US pediatric emergency departments (EDs) differ by race and ethnicity?

**Findings:**

This multicenter cross-sectional study of more than 13 million pediatric ED visits to 44 children’s hospitals demonstrated that non-Hispanic Black and Hispanic patients were less likely to undergo diagnostic imaging compared with non-Hispanic White patients.

**Meaning:**

In these findings, race and ethnicity appear to be independently associated with imaging decisions in the pediatric ED, highlighting the need to better understand and mitigate these disparities.

## Teaser

This cross-sectional study assesses the racial and ethnic differences in the performance of common emergency department imaging studies and examines patterns across diagnoses among US children’s hospitals.

## Introduction

In 2010, the American Academy of Pediatrics published a landmark report highlighting “extensive, pervasive, and persistent” disparities in pediatric health care delivery and quality.^1^^(p1014)^ An important determinant of health care quality is the appropriate use of diagnostic testing for evaluating acute illness in children. In particular, radiologic imaging for pediatric patients is commonly used in the emergency department (ED) setting, with one-third of all visits including at least 1 imaging study.^2^ In addition to the many benefits, imaging also carries risks and considerations regarding resource use, including radiation exposure,^3^ incidental findings leading to follow-up visits and testing,^4^ increased ED length of stay,^5,6^ and cost.^7^ Therefore, differential use of imaging studies across racial and ethnic groups suggests that worse care is being delivered to 1 or more groups.

Studies of racial and ethnic differences in pediatric diagnostic imaging^8,9,10,11,12^ have shown higher rates of selected imaging use in non-Hispanic White children compared with non-White children. However, these studies were limited in scope, focusing on a single imaging modality for a specific condition. One study of ED visits among adults demonstrated that non-Hispanic Black patients were less likely to have radiography, computed tomography (CT), and magnetic resonance imaging (MRI) studies performed.^13^ These patterns in adults may not be relevant for children, because imaging strategies, scope of presenting complaints and diagnoses, and often severity of illness differ between adults and children.^14,15,16^

In our previous work,^6^ we observed that non-Hispanic White children had higher odds of receiving advanced imaging compared with non-White patients. We sought to further explore this finding by evaluating whether racial and ethnic differences exist across imaging modalities and whether these differences persist across diagnoses and by insurance type.

## Methods

### Data Source and Study Design

This multicenter cross-sectional study of the Pediatric Health Information System (PHIS) includes administrative data from 52 tertiary care US children’s hospitals. Participating hospitals are located in 27 states plus Washington, DC, representing 17 of the 20 major metropolitan areas. The Children’s Hospital Association maintains the PHIS and ensures data quality and control through a joint effort with participating hospitals. We included 44 EDs in our study after excluding 8 that did not contribute complete ED data during the study period. We included all ED visits from January 1, 2016, through December 31, 2019, by patients younger than 18 years. This period was selected to enable use of the *International Statistical Classification of Diseases, 10th Revision, Clinical Modification* (*ICD-10-CM*), which was adopted in 2015 across participating sites. This study followed the Strengthening the Reporting of Observational Studies in Epidemiology (STROBE) reporting guideline for cross-sectional studies.^17^ The University of Pittsburgh institutional review board determined that the study protocol was not human subjects research and therefore was exempt from review and informed consent.

### Variables and Outcome Measures

The primary outcome was the proportion of ED visits during which at least 1 diagnostic imaging test, defined as radiography, ultrasonography, CT, and MRI, was performed. These modalities were selected because they represent the most frequently performed diagnostic imaging studies in the emergency setting.^15^ Diagnostic imaging in the PHIS is identified through billing codes and includes the date of imaging. However, for patients who are admitted from the ED, the data source does not distinguish between imaging performed in the ED and imaging performed as an inpatient on the same date. Therefore, and in keeping with prior work,^6,18^ we defined imaging for admitted patients as follows: if ED arrival time was before 6 pm, we attributed imaging to the ED if it occurred on the day of arrival; if ED arrival time was after 6 pm, we attributed imaging to the ED if it occurred on the day of arrival or the next day.

The exposure of interest was documented race and ethnicity. In the PHIS, race and ethnicity are included as 2 distinct variables, which were collapsed into a single variable.^19^ Hospitals submit race and ethnicity data to the PHIS for each visit according to hospital-specific practices, which include parent/guardian self-report at the time of arrival or hospital registration assignment. We categorized race and ethnicity into 4 mutually exclusive groups: non-Hispanic White, non-Hispanic Black, Hispanic of any race, and other.^20^ The category of other included American Indian (0.2%), Asian (2.5%), Native Hawaiian (0.2%), multiracial (1.2%), other race (5.5%), and missing (2.0%). Given the small sample size and heterogeneity of the other group, we focused our analyses on the differences comparing non-Hispanic White patients with non-Hispanic Black and Hispanic patients.

We also analyzed demographic, clinical, and visit covariates that either have been shown to be associated with race/ethnicity and imaging or are part of the behavioral model described by Anderson et al,^21^ a conceptual framework for evaluating and analyzing access and equity in health care, including predisposing, enabling, and need factors. Specifically, we evaluated patient age and sex,^22^ insurance,^23^ time and day of visit,^22,24^ household income,^25,26^ distance from the hospital,^27^ complex chronic conditions,^26^ 3-day revisit,^28,29^ hospitalization (including intensive care unit admission),^26^ visit diagnosis,^13^ and year.^6^ We stratified patient age into clinically meaningful categories (<1, 1-4, 5-12, and 13-17 years) and defined the visit day as weekend vs weekday and arrival time as daytime (8:00 am to 3:59 pm), evening (4:00 to 11:59 pm), or overnight (12:00 to 7:59 am).^30^ Median neighborhood household income, presented as quartiles, was based on patient home 5-digit zip code in the PHIS and mapped to the American Community Survey 5-year data for 2014 to 2018.^31^ Distance to the hospital was based on the distance between the centroids of patient home and hospital 5-digit zip codes. We defined complex chronic conditions using the system-based classification scheme by Feudtner et al,^32^ which has been updated to accommodate *ICD-10-CM* implementation, including neonatal, technology dependence, and organ transplant categories. A visit was considered to be a 3-day revisit if an ED visit occurred within 3 prior calendar days. Given the large number of *ICD-10-CM* codes, we used the major diagnostic category classification system to classify visits into 1 of 26 mutually exclusive major organ system–based categories and thereby define the visit diagnosis.^33^ These diagnostic categories are based on the All Patient Refined–Diagnosis Related Groups classification system, which is based on the principal discharge *ICD-10-CM* diagnosis for the visit^33^ (eTable 1 in the Supplement). As an additional analysis, we also analyzed the top 10 principal *ICD-10-CM* codes responsible for the highest volume of encounters with imaging.

### Statistical Analysis

We summarized data with percentages and used Rao-Scott χ^2^ tests, adjusting for clustering within hospitals, to compare categorical data across race/ethnicity groups. We constructed groups of generalized linear models, including the covariates described above, with a binomial distribution and a random effect for hospital, evaluating the independent association of race/ethnicity on overall and individual imaging modalities (ie, radiography, CT, ultrasonography, and MRI). Because of the strong basis for the multicollinearity among race/ethnicity, insurance type, and median neighborhood income,^34,35^ we performed a variance inflation factor analysis using a cutoff of 5.^36^ For this analysis, income was estimated by race and payer, suggesting the presence of multicollinearity; therefore, we excluded income from all models. Given the large differences in insurance coverage by race and ethnicity^37^ and because of the potential interaction between race and ethnicity and insurance, we replicated the modeling stratified by insurance type. The PHIS does not include data on illness severity (eg, Emergency Severity Index); in addition, non-Hispanic White race may be independently associated with lower^38^ or higher^39^ risk of hospitalization. Therefore, to assess the validity of our findings, we performed a separate analysis in which we limited the cohort to visits by nonhospitalized children.

We used generalized linear modeling (incorporating the covariates described previously) to estimate diagnostic category–specific adjusted odds ratios (aORs) and presented those diagnostic categories that each accounted for at least 0.5% of the total ED cohort as a figure (a complete listing of data for all diagnostic categories is shown in eTable 1 in the Supplement). Finally, we used these models to calculate the adjusted proportion of visits with imaging for each race/ethnicity group. We applied the adjusted proportion of imaging in non-Hispanic White patients to the number of visits by non-Hispanic Black and Hispanic patients. We then calculated the difference in the number of visits with imaging when compared with the adjusted proportion with imaging for non-Hispanic Black and Hispanic patients, thus establishing how many more or fewer visits would have imaging if imaging rates for visits by non-Hispanic Black and Hispanic patients were the same as those for visits by non-Hispanic White patients. Missing data were analyzed as a distinct category for relevant variables. All hypothesis testing was 2-sided, with statistical significance defined as *P* < .05. We used SAS, version 9.4 (SAS Institute, Inc) for all analyses.

## Results

### Characteristics of the Study Cohort

We included a total of 13 087 522 ED visits by 6 230 911 patients (mean [SD] age, 5.8 [5.2] years; 52.7% of visits by male patients and 47.3% by female patients) to the 44 pediatric EDs during the 4-year study period. There were 4 496 961 visits (34.4%) by non-Hispanic White, 3 339 043 (25.5%) by non-Hispanic Black, and 3 722 613 (28.4%) by Hispanic patients in the study cohort and 1 528 905 (11.7%) by patients in the other category (Table 1). Insurance status varied across race/ethnicity groups, with 44.2% of visits by non-Hispanic White patients, 79.5% of visits by non-Hispanic Black patients, 81.6% of visits by Hispanic patients, and 63.4% of visits by patients of other races and ethnicities covered by public insurance (*P* < .001). A higher proportion of non-Hispanic White patients were hospitalized (14.3%) compared with non-Hispanic Black (9.4%), Hispanic (8.2%), and other (10.7%) patients (*P* < .001).

**Table 1.** Demographics of Visits to 44 US Children’s Hospitals, by Race and Ethnicity, 2016-2019^a^

Characteristic	Patient group, No. (%) of visits				
	All (n = 13 087 522 [100])	Non-Hispanic White (n = 4 496 961 [34.4])	Non-Hispanic Black (n = 3 339 043 [25.5])	Hispanic (n = 3 722 613 [28.4])	Other (n = 1 528 905 [11.7])^b^
Imaging					
Any^c^	3 689 163 (28.2)	1 506 178 (33.5)	804 515 (24.1)	970 447 (26.1)	408 023 (26.7)
Radiography	2 946 226 (22.6)	1 178 071 (26.2)	682 166 (20.4)	760 800 (20.4)	325 189 (21.3)
CT	391 519 (3.0)	184 549 (4.1)	81 027 (2.4)	86 856 (2.3)	39 087 (2.6)
Ultrasonography	721 986 (5.5)	306 372 (6.8)	112 230 (3.4)	219 014 (5.9)	84 370 (5.5)
MRI	87 967 (0.7)	45 013 (1.0)	14 862 (0.4)	18 356 (0.5)	9736 (0.6)
Patient demographics					
Age, y					
<1	2 095 046 (16.0)	682 836 (15.2)	535 301 (16.0)	596 037 (16.0)	280 872 (18.4)
1-4	4 568 363 (34.9)	1 492 008 (33.2)	1 171 432 (35.1)	1 321 600 (35.5)	583 323 (38.2)
5-12	4 359 681 (33.3)	1 507 180 (33.5)	1 099 042 (32.9)	1 279 046 (34.4)	474 413 (31.0)
13-17	2 064 432 (15.8)	814 937 (18.1)	533 268 (16.0)	525 930 (14.1)	190 297 (12.4)
Male	6 892 618 (52.7)	2 363 545 (52.6)	1 736 555 (52.0)	1 969 724 (52.9)	822 794 (53.8)
Insurance					
Public	8 434 049 (66.0)	1 959 040 (44.2)	2 632 413 (79.5)	2 888 826 (81.6)	953 770 (63.4)
Private	3 639 416 (28.5)	2 256 483 (50.9)	479 245 (14.5)	451 044 (12.7)	452 644 (30.1)
Other^d^	713 071 (5.6)	215 231 (4.9)	199 869 (6.0)	200 445 (5.7)	97 526 (6.5)
Weekend (vs weekday)	3 773 991 (28.8)	1 356 764 (30.2)	901 966 (27.0)	1 058 157 (28.4)	457 104 (29.9)
ED arrival time					
8:00 am to 3:59 pm	4 908 323 (37.6)	1 666 560 (37.1)	1 332 693 (39.9)	1 357 742 (36.5)	551 328 (36.7)
4:00 to 11:59 pm	6 384 107 (48.9)	2 291 137 (50.9)	1 541 857 (46.2)	1 811 214 (48.7)	739 899 (49.3)
12:00 to 7:59 am	1 760 130 (13.5)	533 564 (11.9)	462 433 (13.8)	553 299 (14.9)	210 834 (14.0)
Median household income					
<25th quartile	2 992 123 (22.9)	466 647 (10.4)	1 271 364 (38.1)	969 112 (26.0)	285 000 (18.6)
25th-50th quartile	2 715 101 (20.7)	777 185 (17.3)	785 451 (23.5)	867 655 (23.3)	284 810 (18.6)
51st-75th quartile	3 264 351 (24.9)	1 200 560 (26.7)	663 973 (19.9)	1 029 831 (27.7)	369 987 (24.2)
>75th quartile	3 873 720 (29.6)	1 957 533 (43.5)	583 059 (17.5)	785 804 (21.1)	547 324 (35.8)
Missing	242 227 (1.9)	95 036 (2.1)	35 196 (1.1)	70 211 (1.9)	41 784 (2.7)
Distance from hospital, miles					
<5	3 436 032 (26.4)	734 924 (16.4)	1 330 054 (39.9)	931 248 (25.1)	439 806 (29.1)
5-10	3 705 634 (28.4)	960 133 (21.4)	1 118 592 (33.5)	1 145 697 (30.9)	481 212 (31.8)
10-20	3 133 697 (24.0)	1 264 780 (28.2)	574 626 (17.2)	940 375 (25.4)	353 916 (23.4)
>20	2 761 265 (21.2)	1 524 896 (34.0)	311 027 (9.3)	686 938 (18.5)	238 404 (15.8)
Clinical characteristics					
Complex chronic conditions	865 089 (6.6)	350 682 (7.8)	215 884 (6.5)	202 190 (5.4)	96 333 (6.3)
3-d ED revisit	144 659 (1.1)	55 387 (1.2)	32 004 (1.0)	40 476 (1.1)	16 792 (1.1)
Hospital admission	1 428 454 (10.9)	642 913 (14.3)	315 129 (9.4)	306 930 (8.2)	163 482 (10.7)
Intensive care unit admission	152 900 (1.2)	65 258 (1.5)	37 553 (1.1)	31 415 (0.8)	18 674 (1.2)
Major diagnostic category					
Alcohol/drug use and induced mental disorders	10 009 (0.1)	4513 (0.1)	1886 (0.1)	2504 (0.1)	1106 (0.1)
Blood and immunological conditions	138 719 (1.1)	38 208 (0.8)	57 925 (1.7)	28 565 (0.8)	14 021 (0.9)
Burns	38 432 (0.3)	13 438 (0.3)	11 556 (0.3)	8081 (0.2)	5357 (0.4)
Circulatory conditions	240 325 (1.8)	89 750 (2.0)	62 528 (1.9)	63 771 (1.7)	24 276 (1.6)
Digestive conditions	1 793 337 (13.7)	627 339 (14.0)	371 817 (11.1)	581 599 (15.6)	212 582 (13.9)
Ear, nose, mouth, and throat conditions	3 252 892 (24.9)	1 016 130 (22.6)	857 565 (25.7)	989 718 (26.6)	389 479 (25.5)
Endocrine and metabolic conditions	171 514 (1.3)	79 310 (1.8)	36 585 (1.1)	37 019 (1.0)	18 600 (1.2)
Eye conditions	331 687 (2.5)	100 806 (2.2)	106 040 (3.2)	87 312 (2.3)	37 529 (2.5)
Female reproductive conditions	65 986 (0.5)	21 401 (0.5)	20 546 (0.6)	17 572 (0.5)	6467 (0.4)
Hepatobiliary and pancreatic conditions	20 440 (0.2)	8125 (0.2)	2852 (0.1)	6983 (0.2)	2480 (0.2)
Human immunodeficiency virus infections	135 (0.001)	16 (0.0004)	90 (0.003)	17 (0.0005)	12 (0.001)
Infectious diseases	1 129 921 (8.6)	332 616 (7.4)	270 248 (8.1)	380 353 (10.2)	146 704 (9.6)
Kidney and urinary conditions	233 346 (1.8)	86 052 (1.9)	43 535 (1.3)	77 254 (2.1)	26 505 (1.7)
Lymphatic, hematopoietic, and other malignancies	11 566 (0.1)	5255 (0.1)	1095 (0.03)	3433 (0.1)	1783 (0.1)
Male reproductive conditions	80 518 (0.6)	29 521 (0.7)	12 716 (0.4)	28 334 (0.8)	9947 (0.7)
Mental health conditions	286 370 (2.2)	141 852 (3.2)	64 840 (1.9)	49 515 (1.3)	30 163 (2.0)
Multiple significant trauma	5161 (0.04)	2572 (0.1)	1186 (0.03)	865 (0.02)	538 (0.04)
Musculoskeletal conditions	1 168 049 (8.9)	483 497 (10.8)	254 183 (7.6)	303 827 (8.2)	126 542 (8.3)
Neonatal conditions	60 363 (0.5)	22 805 (0.5)	11 051 (0.3)	16 196 (0.4)	10 311 (0.7)
Nervous system conditions	581 177 (4.4)	254 540 (5.7)	132 221 (4.0)	133 553 (3.6)	60 863 (4.0)
Poisonings and injuries	279 821 (2.1)	108 261 (2.4)	79 934 (2.4)	59 152 (1.6)	32 474 (2.1)
Pregnancy and childbirth	4991 (0.04)	917 (0.02)	2320 (0.1)	1276 (0.03)	478 (0.03)
Rehabilitation and aftercare	376 599 (2.9)	120 644 (2.7)	97 630 (2.9)	108 626 (2.9)	49 699 (3.3)
Respiratory conditions	1 331 834 (10.2)	401 933 (8.9)	428 398 (12.8)	345 412 (9.3)	156 091 (10.2)
Skin and subcutaneous conditions	1 454 109 (11.1)	500 047 (11.1)	405 058 (12.1)	387 693 (10.4)	161 311 (10.6)
Ungroupable	20 221 (0.2)	7413 (0.2)	5238 (0.2)	3983 (0.1)	3587 (0.2)

Abbreviations: CT, computed tomography; ED, emergency department; MRI, magnetic resonance imaging.

^a^ *P* < .001 (Rao-Scott χ^2^ test) for all variables, except year (*P* = .08).

^b^ Includes American Indian (0.2%), Asian (2.5%), Native Hawaiian (0.2%), multiracial (1.2%), other race (5.5%), and missing (2.0%).

^c^ Indicates number of visits with at least 1 of the 4 imaging modalities performed; sum of each of the 4 imaging modalities is greater than the number of any imaging studies because each visit could include more than 1 imaging modality.

^d^ Includes self-pay (4.5%), charity (0.05%), hospital did not bill (0.003%), and other (0.9%).

### Diagnostic Imaging Rates

A total of 3 689 163 (28.2%) of the 13 087 522 ED visits included 1 or more imaging studies (Table 1). Of these visits with imaging, 79.9% included radiography, 19.6% included ultrasonography, 10.6% included CT, and 2.4% included MRI. More than 1 imaging modality was performed in 339 403 visits (9.2%). Imaging was performed in 33.5% of visits by non-Hispanic White children, compared with 24.1% of visits by non-Hispanic Black children (OR, 0.60; 95% CI, 0.60-0.60) and 26.1% of visits by Hispanic children (OR, 0.66; 95% CI, 0.66-0.67) (Table 2).

**Table 2.** Bivariable and Multivariable Association of Race and Ethnicity With Any Imaging by Insurance Types

Imaging type	For imaging, OR (95% CI)			
	Unadjusted	Adjusted^a^	Adjusted^b^	Adjusted^c^
Any imaging				
Non-Hispanic White	1 [Reference]	1 [Reference]	1 [Reference]	1 [Reference]
Non-Hispanic Black	0.60 (0.60-0.60)	0.82 (0.82-0.83)	0.81 (0.81-0.82)	0.82 (0.82-0.83)
Hispanic	0.66 (0.66-0.67)	0.87 (0.87-0.87)	0.87 (0.86-0.88)	0.87 (0.87-0.88)
Radiography				
Non-Hispanic White	1 [Reference]	1 [Reference]	1 [Reference]	1 [Reference]
Non-Hispanic Black	0.70 (0.70-0.71)	0.90 (0.90-0.91)	0.92 (0.91-0.93)	0.89 (0.88-0.90)
Hispanic	0.72 (0.72-0.72)	0.91 (0.91-0.92)	0.92 (0.91-0.93)	0.91 (0.90-0.91)
CT				
Non-Hispanic White	1 [Reference]	1 [Reference]	1 [Reference]	1 [Reference]
Non-Hispanic Black	0.52 (0.51-0.52)	0.83 (0.82-0.84)	0.79 (0.77-0.80)	0.85 (0.84-0.86)
Hispanic	0.56 (0.56-0.57)	0.86 (0.85-0.87)	0.89 (0.87-0.91)	0.87 (0.85-0.88)
Ultrasonography				
Non-Hispanic White	1 [Reference]	1 [Reference]	1 [Reference]	1 [Reference]
Non-Hispanic Black	0.44 (0.44-0.45)	0.69 (0.68-0.70)	0.68 (0.67-0.69)	0.70 (0.70-0.71)
Hispanic	0.69 (0.68-0.69)	0.86 (0.85-0.89)	0.86 (0.85-0.87)	0.88 (0.87-0.89)
MRI				
Non-Hispanic White	1 [Reference]	1 [Reference]	1 [Reference]	1 [Reference]
Non-Hispanic Black	0.39 (0.38-0.40)	0.83 (0.81-0.85)	0.79 (0.76-0.82)	0.88 (0.85-0.90)
Hispanic	0.43 (0.42-0.44)	0.85 (0.83-0.87)	0.84 (0.80-0.87)	0.89 (0.86-0.92)

Abbreviations: CT, computed tomography; MRI, magnetic resonance imaging; OR, odds ratio.

^a^ Adjusted for age, sex, weekend presentation, hour of presentation, insurance, hospital admission, intensive care unit admission, hospital site, complex chronic conditions, All Patient Refined–Diagnosis Related Group category, year, distance from hospital, and 3-day revisit.

^b^ Visits with public insurance adjusted for all variables included in the adjusted model for all visits, except for insurance.

^c^ Visits with private insurance adjusted for all variables included in the adjusted model for all visits except for insurance.

Adjusting for relevant confounders, visits by non-Hispanic Black (aOR, 0.82; 95% CI, 0.82-0.83) and Hispanic (aOR, 0.87; 95% CI, 0.87-0.87) patients were less likely than those by non-Hispanic White patients to include any imaging (Table 2). These patterns of less imaging use for non-Hispanic Black and Hispanic patients were consistent across individual imaging modalities and persisted when stratified by insurance types (Table 2). Limiting the analysis to the 11 506 168 visits among children discharged from the ED, visits by non-Hispanic Black (aOR, 0.79; 95% CI, 0.79-0.80) and Hispanic (aOR, 0.84; 95% CI, 0.84-0.85) children remained less likely to include imaging compared with visits by non-Hispanic White children (eTable 2 in the Supplement).

### Imaging Across Diagnostic Groups Comparing Visits by Non-Hispanic White With Non-Hispanic Black Patients

Imaging was less likely to be performed during ED visits by non-Hispanic Black patients for 15 of the 26 diagnostic categories (57.7%) (Figure and eTable 3 in the Supplement). For 4 diagnostic categories (skin and subcutaneous conditions [aOR, 1.02; 95% CI, 1.01-1.04], blood and immunological conditions [aOR, 1.08; 95% CI, 1.04-1.12], mental health conditions [aOR, 1.12; 95% CI, 1.07-1.18], and hepatobiliary and pancreatic conditions [aOR, 1.14; 95% CI, 1.01-1.28]), imaging was more likely to be performed during visits by non-Hispanic Black patients, and for 7 diagnostic categories there were no differences. The largest differences in odds of imaging comparing visits by non-Hispanic Black with those by non-Hispanic White patients were for conditions related to the female (aOR, 0.52; 95% CI, 0.49-0.56) and male reproductive system (aOR, 0.58; 95% CI, 0.55-0.62), eyes (aOR, 0.69; 95% CI, 0.65-0.72), and digestive system (aOR, 0.69; 95% CI, 0.69-0.70).

**Figure.**
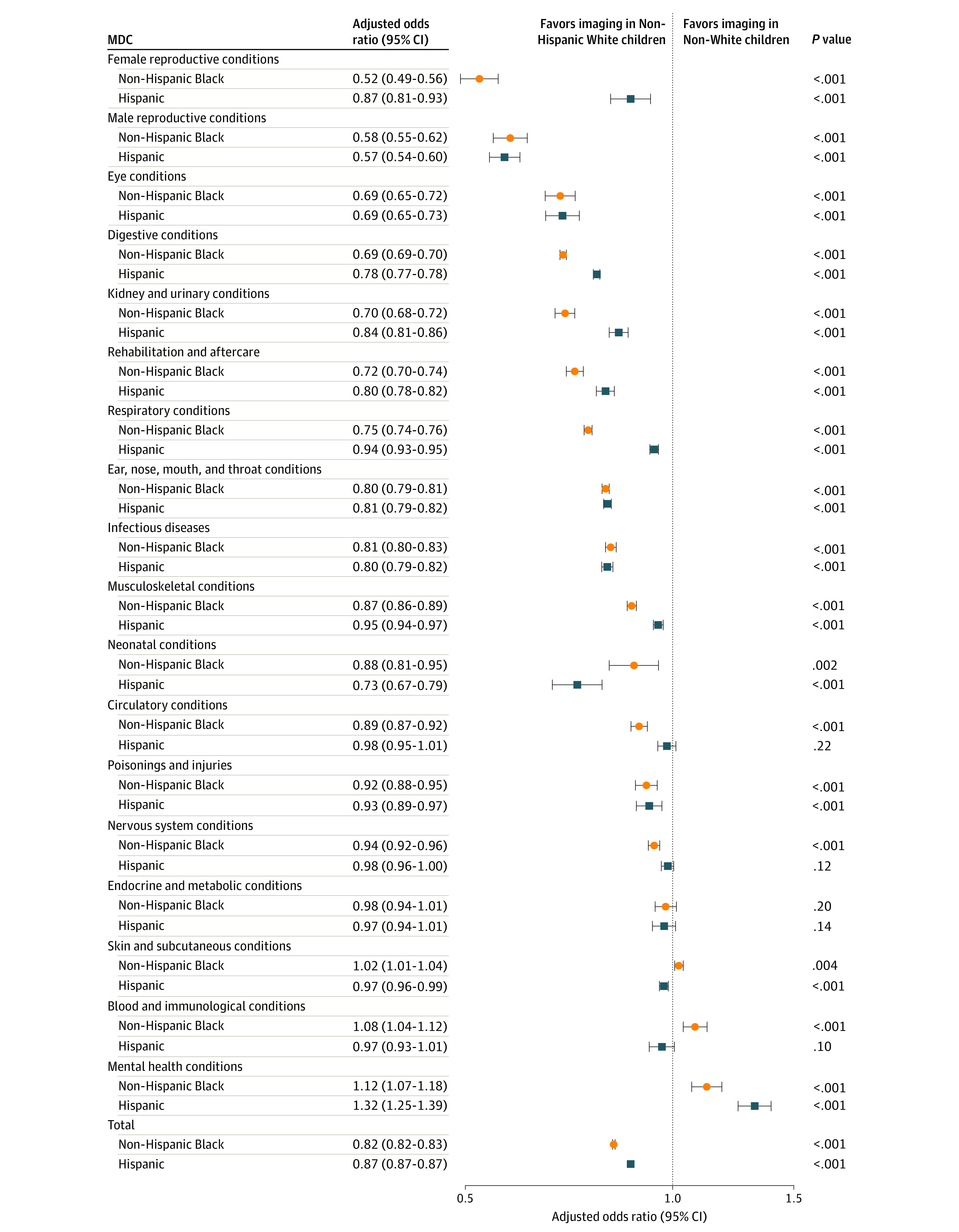
Adjusted Odds of Any Imaging for Visits by Non-Hispanic Black and Hispanic Patients Compared With Non-Hispanic White Patients, by Diagnostic Group Diagnostic categories presented are those that each accounted for at least 0.5% of the total emergency department cohort. MDC indicates major diagnostic category. Odds ratios are adjusted for age, sex, weekend presentation, hour of presentation, insurance, hospital admission, intensive care unit admission, hospital site, complex chronic conditions, year, distance from hospital, and 3-day revisit.

### Imaging Across Diagnostic Groups Comparing Visits by Non-Hispanic White With Hispanic Patients

Imaging was less likely to be performed during visits by Hispanic patients compared with those by non-Hispanic White patients for 13 of the 26 diagnostic categories (50.0%), more likely for 2 diagnostic categories (mental health conditions [aOR, 1.32; 95% CI, 1.25-1.39] and lymphatic, hematopoietic, and other malignancies [malignant neoplasms] [aOR, 1.15; 95% CI, 1.01-1.31), and equallt likely for 11 categories (Figure and eTable 3 in the Supplement). The largest imaging differences were for conditions related to the male reproductive system (aOR, 0.57; 95% CI, 0.54-0.60), eye (aOR, 0.69; 95% CI, 0.65-0.73), and digestive conditions (aOR, 0.78; 95% CI, 0.77-0.78).

Table 3 and eTable 4 in the Supplement show the adjusted differences in the number of visits with imaging by major diagnostic category and *ICD-10-CM* codes, respectively, for non-Hispanic Black and Hispanic patients relative to the expected number of visits with imaging using the adjusted proportion of imaging for non-Hispanic White patients. Overall, if imaging rates among visits by non-Hispanic Black and Hispanic patients were the same as those for visits by non-Hispanic White patients, there would have been 59 993 more visits by non-Hispanic Black patients with imaging and 41 572 more visits by Hispanic patients with imaging. The largest differences were observed for visits related to digestive conditions. Specifically, if the imaging rate for visits by non-Hispanic Black patients with digestive conditions was the same as that for visits by non-Hispanic White patients, there would have been 17 909 (4.8%) more visits with imaging among the 371 817 visits by non-Hispanic Black patients in this diagnostic category; similarly, if the imaging rate for visits by Hispanic patients with digestive conditions was the same as that for visits by non-Hispanic White patients, there would have been 15 067 (2.6%) more visits with imaging among the 581 599 visits by Hispanic patients with this diagnosis.

**Table 3.** Differences in Any Imaging Between Race and Ethnicity Groups by Major Diagnostic Category

Major diagnostic category^a^	Patient group									Adjusted difference in No. of visits with imaging^b^	
	NHW			NHB			Hispanic				
	No. of visits	No. of visits with imaging	Adjusted proportion of visits with imaging, %^c^	No. of visits	No. of visits with imaging	Adjusted proportion of visits with imaging, %^c^	No. of visits	No. of visits with imaging	Adjusted proportion of visits with imaging, %^c^	NHB vs NHW	Hispanic vs NHW
Digestive conditions	627 339	311 700	42.5	371 817	122 990	37.7	581 599	208 132	39.9	−17 909	−15 067
Respiratory conditions	401 933	171 252	36.2	428 398	116 737	32.9	345 412	124 055	35.6	−14 039	−1992
Ear, nose, mouth, and throat conditions	1 016 130	129 733	11.2	857 565	78 389	9.9	989 718	92 937	10.1	−10 909	−10 517
Infectious diseases	332 616	70 661	17.2	270 248	37 486	15.6	380 353	52 585	15.8	−4246	−5327
Musculoskeletal conditions	483 497	396 072	81.1	254 183	202 481	79.7	303 827	241 225	80.7	−3428	−1135
Rehabilitation and aftercare	120 644	27 645	18.7	97 630	13 611	15.8	108 626	15 851	17.0	−2832	−1855
Kidney and urinary conditions	86 052	33 068	33.4	43 535	11 283	28.9	77 254	21 766	31.7	−1952	−1338
Female reproductive conditions	21 401	7214	30.5	20 546	3828	23.7	17 572	5727	29.1	−1402	−245
Eye conditions	100 806	8051	6.1	106 040	4487	5.1	87 312	3875	5.2	−1127	−828
Nervous system conditions	254 540	89 702	32.7	132 221	38 519	31.9	133 553	40 631	32.5	−1088	−205
Circulatory conditions	89 750	44 072	49.7	62 528	29 352	48.1	63 771	32 836	49.5	−1041	−175
Male reproductive conditions	29 521	18 858	56.0	12 716	6024	49.3	28 334	12 394	50.2	−862	−1667
Poisonings and injuries	108 261	15 336	13.3	79 934	10 130	12.8	59 152	6882	12.9	−430	−272
Endocrine and metabolic conditions	79 310	22 342	27.8	36 585	9468	27.5	37 019	10 476	27.5	−99	−110
Neonatal conditions	22 805	4545	16.7	11 051	1556	15.9	16 196	1932	14.6	−91	−342
Pregnancy and childbirth	917	284	28.1	2320	550	26.7	1276	416	28.9	−34	10
Alcohol/drug use and induced mental disorders	4513	607	13.5	1886	237	13.3	2504	383	14.7	−3	31
HIV infections	16	9	57.7	90	52	56.8	17	9	57.1	−1	0
Multiple significant trauma	2572	2406	94.6	1186	1148	94.9	865	834	95.7	4	9
Burns	13 438	673	4.6	11 556	568	4.7	8081	272	4.3	8	−21
Lymphatic, hematopoietic, and other malignancies	5255	3070	57.2	1095	644	58.7	3433	1947	58.7	17	53
Hepatobiliary and pancreatic conditions	8125	5675	69.8	2852	2099	71.5	6983	4859	70.5	48	51
Mental health conditions	141 852	7491	5.5	64 840	3616	5.9	49 515	3679	6.4	237	428
Blood and immunological conditions	38 208	14 678	44.9	57 925	30 561	45.6	28 565	11 023	44.3	397	−173
Skin and subcutaneous conditions	500 047	117 119	20.7	405 058	76 561	20.9	387 693	73 966	20.5	814	−864
Ungroupable	7413	3915	47.2	5238	2138	46.7	3983	1755	46.7	−25	−21

Abbreviations: NHB, non-Hispanic Black; NHW, non-Hispanic White.

^a^ Indicates abbreviated category names (see eTable 1 in the Supplement for full category names).

^b^ Relative to expected number of visits with imaging for NHW patients.

^c^ Adjusted for age, sex, weekend presentation, hour of presentation, insurance, hospital admission, intensive care unit admission, hospital site, complex chronic conditions, All Patient Refined–Diagnosis Related Group category, year, distance from hospital, and 3-day revisit.

## Discussion

In this study of more than 13 million visits to 44 pediatric EDs, we observed that visits by non-Hispanic Black and Hispanic patients were less likely to include radiography, CT, ultrasonography, and MRI compared with those by non-Hispanic White patients. These findings were consistent across most diagnostic groups, persisted when stratified by insurance type, and were even more pronounced on analysis of only visits by nonhospitalized children. Our findings suggest that a child’s race and ethnicity may be independently associated with the decision to perform imaging during ED visits.

The differential use of diagnostic imaging by race/ethnicity may reflect underuse of imaging in non-Hispanic Black and Hispanic children, or alternatively, overuse in non-Hispanic White children. Overuse may expose these children to unnecessary risks associated with imaging.^3,4,7^ Conversely, underuse may result in misdiagnoses, need for further care, and potentially worse clinical outcomes.^40,41,42^ Although we were unable to discern underuse from overuse using an administrative database, it is likely that much of the imaging in White children is unnecessary.^43^ There are many examples of imaging overuse among White children, with no differences in clinical outcomes. For example, compared with non-White children, White children have higher rates of advanced imaging for abdominal pain and abdominal trauma^9,10,44^ and chest radiographs for bronchiolitis,^11^ asthma,^12^ and chest pain.^45^ Similarly, a multicenter study observed that White children with head trauma had higher rates of CT than non-White children,^46^ even among those at the lowest risk for substantial injury.^8^

There are a number of possible explanations for our findings, including a combination of parent/guardian preferences, clinician biases, and structural factors.^47^ Higher imaging rates observed in non-Hispanic White patients may, in part, be attributed to greater levels of parental anxiety with an associated increase in requests for imaging. Such a mechanism has been proposed to be a factor driving the overuse of head imaging in children at low risk of serious traumatic head injury.^8^ There may also be perceived differences in the risk-balance ratio of imaging relative to radiation exposure. A survey of adult patients in the ED reported that White patients preferred a definitive diagnostic test, such as CT, even at the expense of radiation.^48^ Language barriers may also play a role. For example, non–English-speaking patients and their families may be more^49^ or less^24^ likely to have testing performed as part of their ED visit. Physicians’ implicit racial biases are an important consideration and have been associated with patient-clinician interactions, treatment decisions, treatment adherence, and patient health outcomes.^50^ These biases are exacerbated in times of stress, which is particularly relevant to ED clinicians.^51^ Structural factors rooted in our health care system also likely contributed to differential imaging rates. For example, minority patients are less likely than White patients to have a medical home,^52^ which may influence whether clinicians order imaging during the ED visit or defer to outpatient management, and some imaging in White children may have been driven by primary care clinician referral.

With more than 1 in every 4 ED visits in this study including an imaging study, clinicians are frequently performing diagnostic imaging. The goal, undoubtedly, assuming similar clinical presentations across racial and ethnic groups, is to enable parity in diagnostic imaging across these groups. Adherence to clinical guidelines and other objective scoring tools have the potential to reduce subjectivity, support team-based decision-making, and improve communication and structurally competent clinical care.^47,53,54,55^ Internal quality assurance evaluations to better understand physician-level practices that may be influenced by implicit bias may also narrow the disparity gap.^54,56^ In addition, future work is needed to better understand hospital-level disparities in imaging delivery. Such evaluations at the hospital and clinician level are needed to enhance the quality of care delivered and health outcomes for all children.

### Limitations

This study has limitations. The PHIS does not include clinical data regarding the indication for imaging, and there may be unmeasured confounders. We were unable to fully account for illness severity, given the limited clinical information contained within the PHIS (eg, Emergency Severity Index). It is possible that non-Hispanic White children had higher illness acuity, potentially accounting for higher rates of diagnostic imaging. We attempted to minimize this limitation by restricting the analysis to nonhospitalized children and observed even larger differences in imaging rates by race/ethnicity. Race and ethnicity of some patients may have been misclassified given the varying methods of assigning race across PHIS hospitals. However, prior work evaluating race and ethnicity data in children in administrative data^57^ found high accuracy in ethnicity and for White and Black race. We were unable to evaluate or control for limited English proficiency because these data are not available in the PHIS. Imaging for admitted patients may have been misclassified as having occurred as part of the inpatient stay and not the ED visit (and vice versa); notably, admitted patients were a minority of the patient population. Finally, this study was specific to US children’s hospitals, and therefore, the findings are not generalizable to other EDs, care settings, or countries.

## Conclusions

There are significant racial and ethnic differences in diagnostic imaging rates among children seeking care in US pediatric EDs. These differences persist across insurance groups and in analyses limited to discharged children. Further investigation is needed to better understand the factors underpinning these disparities, with the goal of developing measurable interventions to mitigate the disparities in ED imaging and allowing for more equitable and improved care.

## Notes

Supplement. **eTable 1.** Principal *ICD-10-CM* Codes Associated With the Major Diagnostic Categories **eTable 2.** Multivariable Association of Race and Ethnicity With Any Imaging for ED Visits Resulting in Discharge **eTable 3.** Adjusted Odds of Any Imaging for Visits by Non-Hispanic Black and Hispanic Patients Compared With Non-Hispanic White Patients, by Diagnostic Group **eTable 4.** Differences in Any Imaging Between Race and Ethnicity Groups, by Top 10 *ICD-10-CM* Codes With the Highest Volumes of Diagnostic Imaging
