# Supplementary material for: Racial and Ethnic Differences in Emergency Department Diagnostic Imaging at US Children’s Hospitals, 2016-2019
Source: JAMA Netw Open. 2021 Jan 29;4(1):e2033710. doi: 10.1001/jamanetworkopen.2020.33710 (PMC7846940; doi:10.1001/jamanetworkopen.2020.33710)
Supplement: Supplement. — eTable 1. Principal ICD-10-CM Codes Associated With the Major Diagnostic Categories eTable 2. Multivariable Association of Race and Ethnicity With Any Imaging for ED Visits Resulting in Discharge eTable 3. Adjusted Odds of Any Imaging for Visits by Non-Hispanic Black and Hispanic Patients Compared With Non-Hispanic White Patients, by Diagnostic Group eTable 4. Differences in Any Imaging Between Race and Ethnicity Groups, by Top 10 ICD-10-CM Codes With the Highest Volumes of Diagnostic Imaging [file jamanetwopen-e2033710-s001.pdf]

Supplementary Online Content

Marin JR, Rodean J, Hall M, et al. Racial and ethnic differences in emergency department diagnostic imaging at US children's hospitals, 2016-2019. *JAMA Netw Open*. 2021;4(1):e2033710. doi:10.1001/jamanetworkopen.2020.33710

eTable 1. Principal *ICD-10-CM* Codes Associated With the Major Diagnostic Categories

eTable 2. Multivariable Association of Race and Ethnicity With Any Imaging for ED Visits Resulting in Discharge

eTable 3. Adjusted Odds of Any Imaging for Visits by Non-Hispanic Black and Hispanic Patients Compared With Non-Hispanic White Patients, by Diagnostic Group

eTable 4. Differences in Any Imaging Between Race and Ethnicity Groups, by Top 10 *ICD-10-CM* Codes With the Highest Volumes of Diagnostic Imaging

This supplementary material has been provided by the authors to give readers additional information about their work.

©2021 Marin JR et al. *JAMA Network Open*.

eTable 1: Principal ICD10-CM codes associated with the Major Diagnostic Categories

Major Diagnostic Category	ICD10-CM code	ICD10- CM description	Visits, N	% of category[a]
Diseases and disorders of the nervous system	R51	Headache	112,824	19.4
	S060X0A	Concussion without loss of consciousness, initial encounter	61,725	10.6
	R569	Unspecified convulsions	52,715	9.1
	R5600	Simple febrile convulsions	45,643	7.9
	G40909	Epilepsy, unspecified, not intractable, without status epilepticus	29,841	5.1
	G43909	Migraine, unspecified, not intractable, without status migrainosus	26,647	4.6
	S020XXA	Fracture of vault of skull, initial encounter for closed fracture	9,305	1.6
	S060X9A	Concussion with loss of consciousness of unspecified duration, initial encounter	9,046	1.6
	S060X1A	Concussion with loss of consciousness of 30 minutes or less, initial encounter	8,882	1.5
Diseases and disorders of the eye	H109	Unspecified conjunctivitis	43,094	13.0
	H1033	Unspecified acute conjunctivitis, bilateral	25,491	7.7
	S01112A	Laceration without foreign body of left eyelid and periocular area, initial encounter	23,758	7.2
	S01111A	Laceration without foreign body of right eyelid and periocular area, initial encounter	22,777	6.9
	H1032	Unspecified acute conjunctivitis, left eye	11,343	3.4
	H1031	Unspecified acute conjunctivitis, right eye	11,320	3.4
	H1013	Acute atopic conjunctivitis, bilateral	10,382	3.1
	S0501XA	Injury of conjunctiva and corneal abrasion without foreign body, right eye, initial encounter	10,287	3.1
	S0502XA	Injury of conjunctiva and corneal abrasion without foreign body, left eye, initial encounter	9,983	3.0
	H578	Other specified disorders of eye and adnexa	9,534	2.9
	B309	Viral conjunctivitis, unspecified	9,279	2.8
	H10023	Other mucopurulent conjunctivitis, bilateral	9,272	2.8
	H1089	Other conjunctivitis	6,244	1.9
Ear, nose, mouth, throat, and craniofacial diseases and disorders	J069	Acute upper respiratory infection, unspecified	885,508	27.2
	J050	Acute obstructive laryngitis [croup]	229,905	7.1
	J020	Streptococcal pharyngitis	196,178	6.0
	J029	Acute pharyngitis, unspecified	186,387	5.7
	S0990XA	Unspecified injury of head, initial encounter	165,799	5.1
	H6691	Otitis media, unspecified, right ear	124,047	3.8
	H6692	Otitis media, unspecified, left ear	108,078	3.3

©2021 Marin JR et al. *JAMA Network Open*.

	J101	Influenza due to other identified influenza virus with other respiratory manifestations	92,839	2.9
	J111	Influenza due to unidentified influenza virus with other respiratory manifestations	90,778	2.8
	H6693	Otitis media, unspecified, bilateral	78,816	2.4
	H66001	Acute suppurative otitis media without spontaneous rupture of ear drum, right ear	67,861	2.1
	H66002	Acute suppurative otitis media without spontaneous rupture of ear drum, left ear	58,041	1.8
Diseases and disorders of the respiratory system	J219	Acute bronchiolitis, unspecified	204,985	15.4
	J45901	Unspecified asthma with (acute) exacerbation	171,018	12.8
	R05	Cough	152,737	11.5
	J189	Pneumonia, unspecified organism	94,117	7.1
	J210	Acute bronchiolitis due to respiratory syncytial virus	63,187	4.7
	J4521	Mild intermittent asthma with (acute) exacerbation	58,475	4.4
	J45909	Unspecified asthma, uncomplicated	38,930	2.9
	J218	Acute bronchiolitis due to other specified organisms	37,447	2.8
	R062	Wheezing	36,773	2.8
	J209	Acute bronchitis, unspecified	34,626	2.6
	J9601	Acute respiratory failure with hypoxia	29,440	2.2
	J4541	Moderate persistent asthma with (acute) exacerbation	29,202	2.2
	J988	Other specified respiratory disorders	27,208	2.0
	J4531	Mild persistent asthma with (acute) exacerbation	25,185	1.9
	J181	Lobar pneumonia, unspecified organism	20,453	1.5
Diseases and disorders of the circulatory system	R55	Syncope and collapse	54,169	22.5
	R079	Chest pain, unspecified	52,032	21.7
	R0789	Other chest pain	32,546	13.5
	M940	Chondrocostal junction syndrome [Tietze]	25,648	10.7
	R0989	Other specified symptoms and signs involving the circulatory and respiratory systems	12,123	5.0
	R002	Palpitations	6,804	2.8
	R000	Tachycardia, unspecified	5,508	2.3
	I471	Supraventricular tachycardia	3,724	1.5
	R072	Precordial pain	3,489	1.5
Diseases and disorders of the digestive system	R1110	Vomiting, unspecified	290,099	16.2
	K5900	Constipation, unspecified	206,066	11.5
	K529	Noninfective gastroenteritis and colitis, unspecified	195,178	10.9
	A084	Viral intestinal infection, unspecified	118,250	6.6
	R109	Unspecified abdominal pain	113,896	6.4
	R197	Diarrhea, unspecified	94,093	5.2
	R1031	Right lower quadrant pain	55,072	3.1
	R112	Nausea with vomiting, unspecified	54,546	3.0
	R1084	Generalized abdominal pain	53,745	3.0
	K219	Gastro-esophageal reflux disease without esophagitis	39,269	2.2

©2021 Marin JR et al. *JAMA Network Open*.

	R1013	Epigastric pain	33,169	1.8
	R1033	Periumbilical pain	31,414	1.8
Diseases and disorders of the hepatobiliary system and pancreas	K8590	Acute pancreatitis without necrosis or infection, unspecified	2,875	14.1
	K8020	Calculus of gallbladder without cholecystitis without obstruction	2,265	11.1
	R17	Unspecified jaundice	899	4.4
	P599	Neonatal jaundice, unspecified	822	4.0
	K859	Acute pancreatitis, unspecified	670	3.3
	E806	Other disorders of bilirubin metabolism	650	3.2
	K830	Cholangitis	604	3.0
	K861	Other chronic pancreatitis	538	2.6
	K760	Fatty (change of) liver, not elsewhere classified	445	2.2
	P593	Neonatal jaundice from breast milk inhibitor	437	2.1
	K8000	Calculus of gallbladder with acute cholecystitis without obstruction	428	2.1
	K8050	Calculus of bile duct without cholangitis or cholecystitis without obstruction	411	2.0
	K8010	Calculus of gallbladder with chronic cholecystitis without obstruction	397	1.9
	K8500	Idiopathic acute pancreatitis without necrosis or infection	349	1.7
	K8510	Biliary acute pancreatitis without necrosis or infection	322	1.6
	K759	Inflammatory liver disease, unspecified	304	1.5
	Q442	Atresia of bile ducts	298	1.5
Diseases and disorders of the musculoskeletal system and connective tissue	S93401A	Sprain of unspecified ligament of right ankle, initial encounter	31,952	2.7
	S93402A	Sprain of unspecified ligament of left ankle, initial encounter	28,518	2.4
	S53032A	Nursemaid's elbow, left elbow, initial encounter	25,368	2.2
	S6991XA	Unspecified injury of right wrist, hand and finger(s), initial encounter	24,777	2.1
	S6992XA	Unspecified injury of left wrist, hand and finger(s), initial encounter	21,857	1.9
	S52502A	Unspecified fracture of the lower end of left radius, initial encounter for closed fracture	21,853	1.9
	M542	Cervicalgia	19,297	1.7
	M545	Low back pain	17,909	1.5
	S8992XA	Unspecified injury of left lower leg, initial encounter	17,462	1.5
	S42412A	Displaced simple supracondylar fracture without intercondylar fracture of left humerus, initial encounter for closed fracture	17,383	1.5
	S52501A	Unspecified fracture of the lower end of right radius, initial encounter for closed fracture	17,189	1.5
	S8991XA	Unspecified injury of right lower leg, initial encounter	17,169	1.5
Diseases and disorders of the skin,	S0181XA	Laceration without foreign body of other part of head, initial encounter	120,438	8.3
	R21	Rash and other nonspecific skin eruption	93,870	6.5
	L509	Urticaria, unspecified	64,439	4.4

©2021 Marin JR et al. *JAMA Network Open*.

subvutaneous tissue and breast	S0101XA	Laceration without foreign body of scalp, initial encounter	52,905	3.6
	S0083XA	Contusion of other part of head, initial encounter	47,499	3.3
	L0100	Impetigo, unspecified	37,597	2.6
	L309	Dermatitis, unspecified	30,936	2.1
	L22	Diaper dermatitis	26,333	1.8
	S0003XA	Contusion of scalp, initial encounter	21,734	1.5
Endocrine, nutritional and metabolic diseases and disorders	E860	Dehydration	60,347	35.2
	E1010	Type 1 diabetes mellitus with ketoacidosis without coma	18,883	11.0
	E1065	Type 1 diabetes mellitus with hyperglycemia	15,872	9.3
	R633	Feeding difficulties	6,403	3.7
	P9209	Other vomiting of newborn	6,202	3.6
	R6251	Failure to thrive (child)	4,871	2.8
	R638	Other symptoms and signs concerning food and fluid intake	4,626	2.7
	E1165	Type 2 diabetes mellitus with hyperglycemia	3,926	2.3
	E162	Hypoglycemia, unspecified	3,614	2.1
	E109	Type 1 diabetes mellitus without complications	3,017	1.8
	R630	Anorexia	2,978	1.7
Diseases and disorders of the kidney and urinary tract	N390	Urinary tract infection, site not specified	102,873	44.1
	R300	Dysuria	25,549	10.9
	N3000	Acute cystitis without hematuria	12,498	5.4
	N12	Tubulo-interstitial nephritis, not specified as acute or chronic	10,795	4.6
	N10	Acute pyelonephritis	9,898	4.2
	N3001	Acute cystitis with hematuria	7,849	3.4
	R319	Hematuria, unspecified	5,645	2.4
	N200	Calculus of kidney	4,532	1.9
	R350	Frequency of micturition	3,426	1.5
Diseases and disorders of the male reproductive system	N481	Balanitis	11,403	14.2
	N4889	Other specified disorders of penis	7,830	9.7
	N476	Balanoposthitis	5,407	6.7
	N451	Epididymitis	5,337	6.6
	N433	Hydrocele, unspecified	4,864	6.0
	N50812	Left testicular pain	4,755	5.9
	N50811	Right testicular pain	4,576	5.7
	N508	Other specified disorders of male genital organs	3,275	4.1
	N4403	Torsion of appendix testis	3,195	4.0
	N471	Phimosis	2,614	3.2
	N4400	Torsion of testis, unspecified	2,508	3.1
	N5089	Other specified disorders of the male genital organs	2,194	2.7
	N5082	Scrotal pain	2,064	2.6
	N453	Epididymo-orchitis	2,009	2.5
	N50819	Testicular pain, unspecified	1,595	2.0

	N472	Paraphimosis	1,492	1.9
	I861	Scrotal varices	1,329	1.7
	N492	Inflammatory disorders of scrotum	1,276	1.6
Diseases and disorders of the female reproductive system	N760	Acute vaginitis	14,538	22.0
	N898	Other specified noninflammatory disorders of vagina	4,909	7.4
	S3141XA	Laceration without foreign body of vagina and vulva, initial encounter	4,110	6.2
	N946	Dysmenorrhea, unspecified	3,854	5.8
	N939	Abnormal uterine and vaginal bleeding, unspecified	3,836	5.8
	B373	Candidiasis of vulva and vagina	3,525	5.3
	N83201	Unspecified ovarian cyst, right side	3,375	5.1
	N764	Abscess of vulva	3,269	5.0
	N920	Excessive and frequent menstruation with regular cycle	2,011	3.0
	N938	Other specified abnormal uterine and vaginal bleeding	1,948	3.0
	N739	Female pelvic inflammatory disease, unspecified	1,784	2.7
	N83202	Unspecified ovarian cyst, left side	1,688	2.6
	N762	Acute vulvitis	1,215	1.8
	N8320	Unspecified ovarian cysts	1,210	1.8
	N921	Excessive and frequent menstruation with irregular cycle	1,048	1.6
	N9089	Other specified noninflammatory disorders of vulva and perineum	1,013	1.5
Pregnancy, childbirth, and the puerperium	O26891	Other specified pregnancy related conditions, first trimester	530	10.6
	O9989	Other specified diseases and conditions complicating pregnancy, childbirth and the puerperium	486	9.7
	O26892	Other specified pregnancy related conditions, second trimester	313	6.3
	O219	Vomiting of pregnancy, unspecified	305	6.1
	O26899	Other specified pregnancy related conditions, unspecified trimester	235	4.7
	O209	Hemorrhage in early pregnancy, unspecified	202	4.0
	O26893	Other specified pregnancy related conditions, third trimester	171	3.4
	O200	Threatened abortion	166	3.3
	O210	Mild hyperemesis gravidarum	144	2.9
	O039	Complete or unspecified spontaneous abortion without complication	138	2.8
	O2341	Unspecified infection of urinary tract in pregnancy, first trimester	117	2.3
Newborns and other neonates with conditions originating in the perinatal period	P599	Neonatal jaundice, unspecified	19,458	32.2
	P9689	Other specified conditions originating in the perinatal period	3,188	5.3
	P593	Neonatal jaundice from breast milk inhibitor	2,992	5.0
	P809	Hypothermia of newborn, unspecified	1,453	2.4
	P741	Dehydration of newborn	1,136	1.9
	P819	Disturbance of temperature regulation of newborn, unspecified	1,053	1.7

©2021 Marin JR et al. *JAMA Network Open*.

	P598	Neonatal jaundice from other specified causes	1,013	1.7
	P929	Feeding problem of newborn, unspecified	1,009	1.7
	P9209	Other vomiting of newborn	884	1.5
Diseases and disorders of blood, blood forming organs, and immunological disorders	D5700	Hb-SS disease with crisis, unspecified	21,842	15.7
	I889	Nonspecific lymphadenitis, unspecified	14,855	10.7
	R590	Localized enlarged lymph nodes	10,074	7.3
	D709	Neutropenia, unspecified	9,807	7.1
	D571	Sickle-cell disease without crisis	8,347	6.0
	L040	Acute lymphadenitis of face, head and neck	5,954	4.3
	D5701	Hb-SS disease with acute chest syndrome	4,163	3.0
	D693	Immune thrombocytopenic purpura	4,150	3.0
	R591	Generalized enlarged lymph nodes	4,100	3.0
	D509	Iron deficiency anemia, unspecified	3,604	2.6
	D57219	Sickle-cell/Hb-C disease with crisis, unspecified	3,573	2.6
	D701	Agranulocytosis secondary to cancer chemotherapy	3,169	2.3
	R599	Enlarged lymph nodes, unspecified	3,148	2.3
	R233	Spontaneous ecchymoses	2,970	2.1
	D649	Anemia, unspecified	2,964	2.1
	L049	Acute lymphadenitis, unspecified	2,194	1.6
	D696	Thrombocytopenia, unspecified	2,126	1.5
	D57419	Sickle-cell thalassemia with crisis, unspecified	2,023	1.5
Lymphatic, hematopoietic, other malignancies, chemotherapy, and radiotherapy	C9100	Acute lymphoblastic leukemia not having achieved remission	5,745	49.7
	C9101	Acute lymphoblastic leukemia, in remission	1,139	9.8
	Z5111	Encounter for antineoplastic chemotherapy	710	6.1
	C9200	Acute myeloblastic leukemia, not having achieved remission	476	4.1
	C9102	Acute lymphoblastic leukemia, in relapse	350	3.0
Infectious and parasitic diseases, systemic or unspecified sites	R509	Fever, unspecified	521,220	46.1
	B349	Viral infection, unspecified	345,636	30.6
	B084	Enteroviral vesicular stomatitis with exanthem	83,558	7.4
	B09	Unspecified viral infection characterized by skin and mucous membrane lesions	47,487	4.2
Mental diseases and disorders	F329	Major depressive disorder, single episode, unspecified	47,985	16.8
	R45851	Suicidal ideations	41,777	14.6
	R4689	Other symptoms and signs involving appearance and behavior	16,893	5.9
	F419	Anxiety disorder, unspecified	15,684	5.5
	F919	Conduct disorder, unspecified	12,143	4.2
	F3481	Disruptive mood dysregulation disorder	8,388	2.9
	F39	Unspecified mood [affective] disorder	7,137	2.5
	F332	Major depressive disorder, recurrent severe without psychotic features	6,988	2.4
	F911	Conduct disorder, childhood-onset type	6,503	2.3
	F410	Panic disorder [episodic paroxysmal anxiety]	6,159	2.2

©2021 Marin JR et al. *JAMA Network Open*.

	F909	Attention-deficit hyperactivity disorder, unspecified type	5,333	1.9
	F411	Generalized anxiety disorder	4,797	1.7
	F918	Other conduct disorders	4,773	1.7
	F4310	Post-traumatic stress disorder, unspecified	4,659	1.6
	F913	Oppositional defiant disorder	4,594	1.6
	R4589	Other symptoms and signs involving emotional state	4,463	1.6
	F840	Autistic disorder	4,402	1.5
	F322	Major depressive disorder, single episode, severe without psychotic features	4,320	1.5
Alcohol/drug use and alcohol/drug-induced organic mental disorders	F10129	Alcohol abuse with intoxication, unspecified	1,953	19.5
	F1210	Cannabis abuse, uncomplicated	913	9.1
	F1910	Other psychoactive substance abuse, uncomplicated	736	7.4
	F1290	Cannabis use, unspecified, uncomplicated	728	7.3
	F10120	Alcohol abuse with intoxication, uncomplicated	611	6.1
	F12929	Cannabis use, unspecified with intoxication, unspecified	567	5.7
	F10920	Alcohol use, unspecified with intoxication, uncomplicated	390	3.9
	F10929	Alcohol use, unspecified with intoxication, unspecified	366	3.7
	F12920	Cannabis use, unspecified with intoxication, uncomplicated	257	2.6
	F1990	Other psychoactive substance use, unspecified, uncomplicated	239	2.4
	F1310	Sedative, hypnotic or anxiolytic abuse, uncomplicated	204	2.0
	F12129	Cannabis abuse with intoxication, unspecified	180	1.8
	F1010	Alcohol abuse, uncomplicated	168	1.7
Poisonings, toxic effects, other injuries, and other complications of treatment	T7840XA	Allergy, unspecified, initial encounter	28,738	10.3
	Z041	Encounter for examination and observation following transport accident	27,434	9.8
	T7622XA	Child sexual abuse, suspected, initial encounter	15,914	5.7
	Z043	Encounter for examination and observation following other accident	14,130	5.0
	T7612XA	Child physical abuse, suspected, initial encounter	10,143	3.6
	T782XXA	Anaphylactic shock, unspecified, initial encounter	10,136	3.6
	T781XXA	Other adverse food reactions, not elsewhere classified, initial encounter	9,765	3.5
	T7422XA	Child sexual abuse, confirmed, initial encounter	6,771	2.4
	J95830	Postprocedural hemorrhage of a respiratory system organ or structure following a respiratory system procedure	6,382	2.3
	T63441A	Toxic effect of venom of bees, accidental (unintentional), initial encounter	5,679	2.0
	T751XXA	Unspecified effects of drowning and nonfatal submersion, initial encounter	4,428	1.6
	T7412XA	Child physical abuse, confirmed, initial encounter	4,166	1.5
Burns	T2121XA	Burn of second degree of chest wall, initial encounter	2,375	6.2
	T2122XA	Burn of second degree of abdominal wall, initial encounter	1,406	3.7
	T23252A	Burn of second degree of left palm, initial encounter	1,305	3.4

©2021 Marin JR et al. *JAMA Network Open*.

	T24212A	Burn of second degree of left thigh, initial encounter	1,178	3.1
	T24211A	Burn of second degree of right thigh, initial encounter	1,117	2.9
	T23251A	Burn of second degree of right palm, initial encounter	1,112	2.9
	T25222A	Burn of second degree of left foot, initial encounter	935	2.4
	T23201A	Burn of second degree of right hand, unspecified site, initial encounter	922	2.4
	T22212A	Burn of second degree of left forearm, initial encounter	917	2.4
	T22211A	Burn of second degree of right forearm, initial encounter	911	2.4
	T25221A	Burn of second degree of right foot, initial encounter	878	2.3
	T23202A	Burn of second degree of left hand, unspecified site, initial encounter	848	2.2
	T2026XA	Burn of second degree of forehead and cheek, initial encounter	775	2.0
	T2020XA	Burn of second degree of head, face, and neck, unspecified site, initial encounter	738	1.9
	T23231A	Burn of second degree of multiple right fingers (nail), not including thumb, initial encounter	709	1.8
	T23232A	Burn of second degree of multiple left fingers (nail), not including thumb, initial encounter	655	1.7
	T2220XA	Burn of second degree of shoulder and upper limb, except wrist and hand, unspecified site, initial encounter	610	1.6
Rehabilitation, aftercare, other factors influencing health status and other health service contacts	Z5321	Procedure and treatment not carried out due to patient leaving prior to being seen by health care provider	72,497	19.3
	R6812	Fussy infant (baby)	28,546	7.6
	Z711	Person with feared health complaint in whom no diagnosis is made	17,040	4.5
	P9689	Other specified conditions originating in the perinatal period	12,698	3.4
	Z0389	Encounter for observation for other suspected diseases and conditions ruled out	12,370	3.3
	R69	Illness, unspecified	11,655	3.1
	G8918	Other acute postprocedural pain	8,612	2.3
	R4182	Altered mental status, unspecified	8,544	2.3
	Z4802	Encounter for removal of sutures	8,317	2.2
	R6813	Apparent life threatening event in infant (ALTE)	8,057	2.1
	Z23	Encounter for immunization	7,190	1.9
	R6889	Other general symptoms and signs	7,016	1.9
	Z0442	Encounter for examination and observation following alleged child rape	6,548	1.7
	Z4789	Encounter for other orthopedic aftercare	5,649	1.5
	R6811	Excessive crying of infant (baby)	5,609	1.5
	Z029	Encounter for administrative examinations, unspecified	5,495	1.5
Human immunodeficiency	B20	Human immunodeficiency virus disease	76	56.3
	J189	Pneumonia, unspecified organism	8	5.9
	A419	Sepsis, unspecified organism	5	3.7

©2021 Marin JR et al. *JAMA Network Open*.

virus [HIV] infections	K529	Noninfective gastroenteritis and colitis, unspecified	5	3.7
	G40909	Epilepsy, unspecified, not intractable, without status epilepticus	3	2.2
	L03115	Cellulitis of right lower limb	2	1.5
	L02416	Cutaneous abscess of left lower limb	2	1.5
	L02411	Cutaneous abscess of right axilla	2	1.5
	J159	Unspecified bacterial pneumonia	2	1.5
	A0839	Other viral enteritis	2	1.5
	R21	Rash and other nonspecific skin eruption	2	1.5
Multiple significant trauma	S36116A	Major laceration of liver, initial encounter	218	4.2
	S065X9A	Traumatic subdural hemorrhage with loss of consciousness of unspecified duration, initial encounter	206	4.0
	S36031A	Moderate laceration of spleen, initial encounter	151	2.9
	S066X9A	Traumatic subarachnoid hemorrhage with loss of consciousness of unspecified duration, initial encounter	132	2.6
	S020XXA	Fracture of vault of skull, initial encounter for closed fracture	132	2.6
	S062X9A	Diffuse traumatic brain injury with loss of consciousness of unspecified duration, initial encounter	125	2.4
	S36115A	Moderate laceration of liver, initial encounter	115	2.2
	S36032A	Major laceration of spleen, initial encounter	112	2.2
	S36113A	Laceration of liver, unspecified degree, initial encounter	100	1.9
	S270XXA	Traumatic pneumothorax, initial encounter	99	1.9
	S065X0A	Traumatic subdural hemorrhage without loss of consciousness, initial encounter	85	1.6

[a]ICD10-CM codes responsible for at least 1.5% of visits in the Major Diagnostic Category are shown
ICD10-CM= International Classification of Diseases, 10th Revision, Clinical Modification

©2021 Marin JR et al. *JAMA Network Open*.

eTable 2: Multivariable Association of Race and Ethnicity with Any Imaging for ED Visits Resulting in Discharge

	**Adjusted[a] OR for imaging (95%CI)**
Any imaging	
Non-Hispanic White	Reference
Non-Hispanic Black	0.79 (0.79, 0.80)
Hispanic	0.84 (0.84, 0.85)
XR	
Non-Hispanic White	Reference
Non-Hispanic Black	0.88 (0.88, 0.88)
Hispanic	0.90 (0.89, 0.90)
CT	
Non-Hispanic White	Reference
Non-Hispanic Black	0.72 (0.71, 0.73)
Hispanic	0.79 (0.78, 0.80)
Ultrasound	
Non-Hispanic White	Reference
Non-Hispanic Black	0.62 (0.61, 0.62)
Hispanic	0.79 (0.78, 0.80)
MRI	
Non-Hispanic White	Reference
Non-Hispanic Black	0.66 (0.63, 0.68)
Hispanic	0.71 (0.68, 0.73)

[a]Adjusted for: age, sex, weekend presentation, hour presentation, insurance, intensive care unit admission, hospital site, complex chronic conditions, APR-DRG category, year, distance from hospital, and 3-day revisit
OR=odds ratio, CI=confidence interval, XR= x-ray, CT=computed tomography, MRI= magnetic resonance imaging

©2021 Marin JR et al. *JAMA Network Open*.

eTable 3: Adjusted Odds of Any Imaging for Visits by Non-Hispanic Black and Hispanic Patients Compared with Non-Hispanic White Patients, by Diagnostic Group

Major Diagnostic Category[a]	Adjusted[b] OR for imaging, Non-Hispanic Black[c]	95% CI		Adjusted[b] OR for imaging, Hispanic[c]	95% CI	
Female reproductive conditions	0.52	0.49	0.56	0.87	0.81	0.93
Male reproductive conditions	0.58	0.55	0.62	0.57	0.54	0.60
Eye conditions	0.69	0.65	0.72	0.69	0.65	0.73
Digestive conditions	0.69	0.69	0.70	0.78	0.77	0.78
Kidney and urinary conditions	0.70	0.68	0.72	0.84	0.81	0.86
Rehabilitation and aftercare	0.72	0.70	0.74	0.80	0.78	0.82
Respiratory conditions	0.75	0.74	0.76	0.94	0.93	0.95
Pregnancy and childbirth	0.75	0.59	0.96	1.09	0.84	1.43
Ear, nose, mouth, throat disorders	0.80	0.79	0.81	0.81	0.79	0.82
Infectious diseases	0.81	0.80	0.83	0.80	0.79	0.82
Musculoskeletal conditions	0.87	0.86	0.89	0.95	0.94	0.97
Neonatal conditions	0.88	0.81	0.95	0.73	0.67	0.79
Circulatory conditions	0.89	0.87	0.92	0.98	0.95	1.01
Poisonings and injuries	0.92	0.88	0.95	0.93	0.89	0.97
Human immunodeficiency virus infections	0.92	0.18	4.79	0.95	0.11	8.13
Nervous system conditions	0.94	0.92	0.96	0.98	0.96	1.00
Endocrine and metabolic conditions	0.98	0.94	1.01	0.97	0.94	1.01
Alcohol/drug use and induced mental disorders	0.99	0.81	1.20	1.20	0.99	1.45
Burns	1.02	0.88	1.18	0.91	0.76	1.10
Skin and subcutaneous conditions	1.02	1.01	1.04	0.97	0.96	0.99
Blood and immunological conditions	1.08	1.04	1.12	0.97	0.93	1.01
Multiple significant trauma	1.11	0.72	1.70	1.42	0.89	2.27
Mental diseases and disorders	1.12	1.07	1.18	1.32	1.25	1.39
Lymphatic, hematopoietic and other malignancies	1.13	0.95	1.35	1.15	1.01	1.31
Hepatobiliary and pancreatic conditions	1.14	1.01	1.28	1.07	0.97	1.19
Ungroupable	0.95	0.85	1.07	0.95	0.83	1.07

[a]Abbreviated category names (see eTable 1 for full category names)
[b]Adjusted for: age, sex, weekend presentation, hour presentation, insurance, hospital admission, intensive care unit admission, hospital site, complex chronic conditions, year, distance from hospital, and 3-day revisit
[c]Compared with visits by non-Hispanic White patients
OR=odds ratio, CI=confidence interval

©2021 Marin JR et al. *JAMA Network Open*.

eTable 4: Differences in Any Imaging Between Race and Ethnicity Groups, by Top Ten ICD10-CM Codes with the Highest Volumes of Diagnostic Imaging

	All visits			NHW			NHB			Hispanic			NHB vs. NHW	Hispanic vs NHW
**ICD10-CM principal diagnosis code**	**Visits with imaging, N**	**Visits, N**	**Proportion of visits with imaging**	**Visits with imaging, N**	**Visits, N**	**Adjusted proportion[a] of visits with imaging, %**	**Visits with imaging, N**	**Visits, N**	**Adjusted proportion[a] of visits with imaging, %**	**Visits with imaging, N**	**Visits, N**	**Adjusted proportion[a] of visits with imaging, %**	**Adjusted difference in number of visits with imaging[b]**	
Constipation	116,743	183,452	63.6%	52,978	72,373	65.8%	27,421	46,459	61.0%	36,344	64,620	62.6%	-2240	-2098
Acute upper respiratory infection	96,082	772,429	12.4%	35,257	225,707	13.6%	26,135	248,012	11.4%	34,690	298,710	12.2%	-5470	-4151
Fever	74,893	455,121	16.5%	31,604	152,639	17.3%	17,419	113,020	16.1%	25,870	189,462	15.7%	-1298	-2933
Pneumonia	65,010	80,400	80.9%	27,357	33,954	80.7%	15,813	19,249	81.0%	21,840	27,197	81.0%	57	94
Abdominal pain	58,041	100,138	58.0%	28,527	44,381	59.7%	10,156	19,821	53.9%	19,358	35,936	57.7%	-1162	-726
Vomiting	52,928	253,257	20.9%	24,316	83,026	22.8%	12,002	65,552	19.3%	16,610	104,679	19.6%	-2294	-3414
Viral infection	44,703	299,454	14.9%	18,317	96,165	16.1%	11,627	94,214	13.7%	14,759	109,075	14.6%	-2234	-1674
Right lower quadrant pain	42,814	49,498	86.5%	23,428	26,873	87.1%	5098	6091	84.6%	14,288	16,534	86.2%	-150	-145
Acute bronchiolitis	38,683	179,856	21.5%	15,844	67,342	22.1%	10,764	57,421	20.3%	12,075	55,093	21.8%	-1031	-157
Cough	34,539	133,906	25.8%	14,077	44,338	28.0%	9050	43,180	22.8%	11,412	46,388	25.9%	-2249	-966

[a]Adjusted for: age, sex, weekend presentation, hour presentation, insurance, hospital admission, intensive care unit admission, hospital site, complex chronic conditions, year, distance from hospital, and 3-day revisit
[b]Relative to expected number of visits with imaging for NHW patients
NHW=non-Hispanic White, NHB= non-Hispanic Black, ICD10-CM= *International Classification of Diseases, 10th revision, Clinical Modification*

©2021 Marin JR et al. *JAMA Network Open*.
